# Serum Concentrations of Selected Biological Factors as a Potential Tool for Detecting Recurrence in Endocrine Tumors—A Pilot Study

**DOI:** 10.3390/jcm14113732

**Published:** 2025-05-26

**Authors:** Anna Kurzynska, Elwira Przybylik-Mazurek, Karolina Morawiec-Slawek, Magdalena Kolasa, Edyta Tkacz, Agnieszka Stefanska, Małgorzata Szuminska, Anna Sowa-Staszczak, Justyna Brodowicz, Katarzyna Gawlik, Dorota Pawlica-Gosiewska, Bogdan Solnica, Alicja Hubalewska-Dydejczyk, Marta Opalinska

**Affiliations:** 1 Chair and Department of Endocrinology, Jagiellonian University Medical College, Jakubowskiego str. 2, 30-688 Kraków, Poland; a.kurzynska@uj.edu.pl (A.K.); karolina.morawiec-slawek@uj.edu.pl (K.M.-S.); anna.sowa-staszczak@uj.edu.pl (A.S.-S.); alahub@cm-uj.krakow.pl (A.H.-D.); 2 Department of Endocrinology, Oncological Endocrinology, Nuclear Medicine and Internal Medicine, University Hospital in Krakow, Jakubowskiego str. 2, 30-688 Kraków, Poland; mkolasa@su.krakow.pl (M.K.); erodzik@su.krakow.pl (E.T.); astefanska@su.krakow.pl (A.S.); mszuminska@su.krakow.pl (M.S.); 3 Department of Clinical Biochemistry, Jagiellonian University Medical College, Skawinska str. 8, 31-066 Kraków, Poland; justyna.brodowicz@uj.edu.pl (J.B.); k.gawlik@uj.edu.pl (K.G.); dorota.pawlica@uj.edu.pl (D.P.-G.); bogdan.solnica@uj.edu.pl (B.S.)

**Keywords:** NET, endocrine neoplasms, Fascin, TNF-α, reccurence risk, growth factors

## Abstract

**Objectives:** The current standard of care for endocrine tumors includes a personalized diagnostic and therapeutic approach aimed at the early detection of tumor recurrence after radical surgery. Assessment of tumor-associated biological factors in serum may be useful in patient management. The aim of this study is to determine whether any of the selected growth factors (VEGF, FGF), lectins (Galectin-1, Galectin-3), proteins (Fascin), or TNF-α measured in serum may serve as a potential marker of recurrence. **Methods:** A total of 68 cases, including 43 patients with disseminated endocrine neoplasm (neuroendocrine tumor (NET) 30 cases, medullary thyroid cancer (MTC) 6 cases, adrenal neoplasm 7 cases) and 25 healthy participants, were included in the analysis. Serum concentrations of TNF-α, Fascin, VEGF, Galectin-1, Galectin-3, and FGF were determined in all cases. The results were compared between groups. **Results:** A comparison between all patients and controls revealed differences in TNF-α concentrations (2.88 vs. 0.93 (ng/mL), *p* = 0.008). When comparing the concentrations of the measured factors between the subgroups (classified by tumor type) and the control group, differences were found for TNF-α (*p* = 0.007) and Fascin (*p* = 0.035). In the case of Fascin, differences were found for MTC and adrenal neoplasm patients (0.52 vs. 5.28 (ng/mL), *p* = 0.048), as well as MTC and NET patients (0.52 vs. 5.59 (ng/mL), *p* = 0.007), while the differences between NET patients and controls were close to significance (5.59 vs. 3.67 (ng/mL), *p* = 0.076). For TNF-α, significant differences were found between NET patients and controls (2.88 vs. 0.03 (ng/mL), *p* = 0.005) as well as between MTC patients and controls (2.77 vs. 0.93 (ng/mL), *p* = 0.004). **Conclusions:** Serum concentrations of selected proteins and growth factors (Fascin, TNF-α) are significantly higher in those with disseminated endocrine tumors compared to healthy controls. More studies are needed to determine the role of these selected proteins and growth factors in the early detection of NET/MTC recurrence.

## 1. Introduction

The current standard of care for patients with endocrine neoplasms emphasizes personalized diagnostic and therapeutic approaches. Several critical time points are essential in the management of these patients, particularly early detection of recurrence following radical surgery, monitoring response to therapy, and disease progression. Relapse of hormone-producing neuroendocrine tumors (NETs) (e.g., insulinomas, glucagonomas), medullary thyroid cancer (MTC), or hormonally active adrenal neoplasms can generally be diagnosed by specific monoanalytical tests [1]. In contrast, non-functional tumors must be monitored with anatomical or functional imaging due to the lack of reliable tumor markers. It increases the cost of follow-up and often exposes the patient to radiation.

Among hormonally inactive tumors, excluding MTC, for which calcitonin is the primary marker, only NETs have established biomarkers. However, chromogranin A (CgA) [2] and neuron-specific enolase, routinely used in patients with NET, do not provide the desired sensitivity and specificity. In recent years, there has been increasing interest in the potential application of circulating tumor cells [3] and multianalytical approaches, such as circulating microRNAs and the NETest [4]. These methods demonstrate better diagnostic performance than traditional biomarkers. Unfortunately, their availability is limited, and they are not yet part of routine clinical practice.

In light of this, ongoing research continues to explore potential biomarkers for endocrine neoplasia, particularly those that are potentially available and may hold significant promise for future clinical use. Certain soluble biomarkers, including cytokines and growth factors that are involved in tumorigenesis and are overexpressed in tumor tissues, have previously shown to serve as valuable markers to detect the recurrence of endocrine neoplasms [5,6,7,8], but data are scarce. A 2022 systematic review to identify relevant circulating biomarkers of angiogenesis in neuroendocrine tumors found only 11 articles suitable for analysis. No solid evidence was found to support the use of angiogenic biomarkers in routine practice, although the results show promising directions [9]. For this reason, the choice of biomarkers for this study was not obvious and resulted from the data available in the literature for the other neoplasms.

The aim of this study was to investigate whether selected growth factors (VEGF, FGF), lectins (Galectin-1, Galectin-3), protein (Fascin), or cytokine (TNF-α) measured in the serum of patients with disseminated endocrine neoplasms could serve as potential biomarkers for active disease or tumor recurrence.

## 2. Materials and Methods

### 2.1. Study Population and Design

This was a prospective study conducted at a single university center. The study included patients treated at the Department of Endocrinology, Oncological Endocrinology, Nuclear Medicine and Internal Medicine, University Hospital in Krakow. In all patients pathologically confirmed endocrine neoplasms (NET, MTC, adrenal npl) and dissemination to distant organs was diagnosed by appropriate imaging studies. All were treated with systemic therapies according to oncological standards. The control group consisted of healthy individuals with no current or previous history of oncological or inflammatory systemic diseases. Exclusion criteria included the use of immunosuppressive drugs, chronic inflammatory conditions, autoimmune diseases, or any other disorders that could affect biochemical test results. Their health status was confirmed through medical interviews and available medical documentation.

### 2.2. Data Collection and Determination of Serum Biochemical Factor Concentration

Data collection was performed during routine control visits to the outpatient clinic. The clinical data were obtained from an electronic database. Blood was collected for biochemical tests according to the internal standard. Fasting venous blood samples were taken in the morning, in the case of NET patients, before long-acting somatostatin analog (SSA) administration. Anticoagulant-free vacuum blood collection tubes were used to collect blood samples. After collection, the tubes were left undisturbed to coagulate for 30 min at room temperature. Following this interval, the coagulated blood was centrifuged at 3000 revolutions per minute for 15 min. The serum supernatant was aspirated using a laboratory pipette, transferred to Eppendorf Safe-Lock Tubes (Eppendorf, Hamburg, Germany) with a volume capacity of 1.5 mL in 300 µL aliquots, and stored at a temperature of −80 °C until needed for further analysis. The concentrations of TNF-α, VEGF, FGF, Fascin, Galectin-1, and Galectin-3 in the serum samples were manually quantified using the enzyme-linked immunosorbent assay (ELISA) method with Quantikine ELISA Kits (R&D Systems, Bio-Techne, Minneapolis, MN, USA), following the protocol supplied by the manufacturer. The sensitivity of the assay was 0.004 ng/mL for TNF-α, 0.009 ng/mL for VEGF, 0.003 ng/mL for FGF, 0.47 ng/mL for Fascin, 0.022 ng/mL for Galectin-1, and 0.016 ng/mL for Galectin-3. The intra-assay and interassay coefficients of variation (CV%) for TNF-α, VEGF, FGF, Fascin, Galectin-1, and Galectin-3 were as follows: 2.6% and 7.7%, 5.43% and 7.33%, 5.27% and 8.03%, 5.09% and 5.22%, 7.2% and 8.6%, 3.67% and 6.1%, respectively. The optical density of the samples was measured immediately at 450 nm with a reference wavelength of 565 nm using the BioTek 800 TS absorbance reader (BioTek Instruments, Winooski, VT, USA). Data were collected using the onboard BioTek Gen5 software (BioTek Instruments, Winooski, VT, USA). The standard curve was generated using four-parameter logistic (4PL) curve fitting. The concentrations of TNF-α, VEGF, FGF, Fascin, Galectin-1, and Galectin-3 were expressed in ng/mL. The laboratory analyst performing the biochemical analyses was blinded to the participants’ diagnosis to minimize systematic errors.

### 2.3. Statistical Analysis

The analysis was carried out using IBM SPSS Statistics for Windows, version 29.0.2.0 (IBM Corp., Armonk, NY, USA). Demographic and clinical characteristics were assessed by generating frequency tables for categorical variables and calculating the median and range for continuous variables, given the relatively small sample size and predominantly non-normal data distribution. Categorical variables were reported as counts (*n*) and percentages (%). Statistical comparisons between patient groups were carried out using nonparametric tests, with the Wilcoxon rank sum test applied for continuous variables. For comparisons involving more than two groups, the Kruskal–Wallis test was used. All tests were two-tailed, with a significance level set at α = 0.05 and *p* values < 0.05.

### 2.4. Baseline Patients Characteristics

A total of 68 cases, including 43 patients with disseminated endocrine neoplasms (30 with NET, including 6 patients with hormonally active NETs, and 24 with inactive ones, 6 with MTC, and 7 with adrenal neoplasms) and 25 healthy control participants, were included in the analysis. The mean age of the patients with endocrine neoplasm and the healthy control group was 55.2 years (range 22–89) and 55.0 years (range 26–78), respectively. In both groups, there was a predominance of females: 61% in the endocrine neoplasm group and 84% in the control group. In the NET group, 33% had a G1 tumor and the mean Ki67 index was 6.1% (range 1–20%). Among the NET patients, 12 were diagnosed with pancreatic NET (panNET), 11 with small intestinal (siNET), and 7 with other types of NET (rectum, stomach, lung). The detailed data have been presented in Table 1. Values of CgA routinely assessed in NET patients were negative (in normal range) in 18 cases and positive (over an upper normal range) in 12 cases, mainly in patients with siNET. Median CgA was 5.4, range 2–86.6 nmol/L (normal range 0–6.8). The median values of all the factors measured for both the study and healthy control groups are presented in Table 2. A difference was found between the endocrine neoplasm and the healthy control only in the concentration of TNF-α (*p* = 0.008).

A model comparing the differences between the disease subgroups (NET, MTC, adrenals) and the controls revealed significance for Fascin (*p* = 0.035) and TNF-α concentrations (*p* = 0.007, Table 2). For Fascin, significant differences were observed when comparing patients with MTC with those with adrenal neoplasms (*p* = 0.048) and NETs (*p* = 0.007), while the differences between patients with NET and controls, as well as between patients with MTC and controls, approached significance (*p* = 0.083).

Significant differences in TNF-α concentrations were observed when comparing NET patients and MTC with controls (*p* = 0.004 and *p* = 0.005, respectively). The median values and ranges of TNF-alpha and Fascin concentrations in the respective study groups are presented in Table 3.

### 2.5. Analysis in the NET Group

#### 2.5.1. Effect of Somatostatin Analog Type on Assay Results

All NET patients were treated with long-acting somatostatin analogs administered in standard doses (30 mg of octreotide or 120 mg of lanreotide) every 4 weeks. Significant differences in VEGF concentrations were observed (Table 4), with higher concentrations in patients treated with octreotide. The concentrations of the other parameters evaluated did not differ between the groups.

#### 2.5.2. Impact of Primary Focus Location and NET Grading on the Assays Results

**Figure 1:**
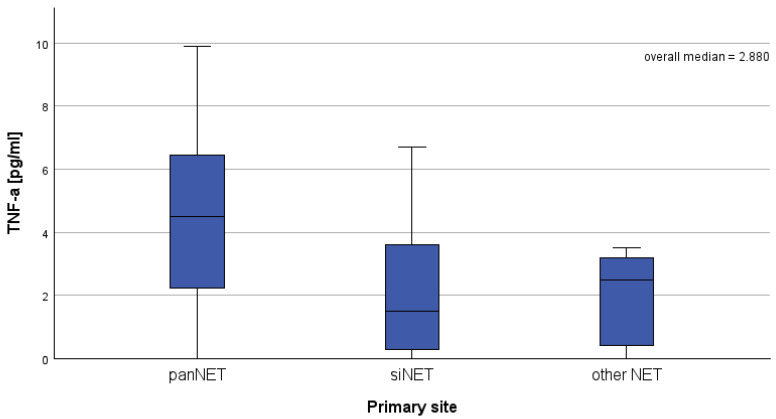
Comparison of serum TNF-alpha concentrations according to the location of the primary NET site, *p* = 0.040.

Significant differences in serum TNF-α concentrations were observed depending on the primary focus of the disease (panNET vs. siNET vs. other locations), with the highest values found in patients with panNET (Table 5, Figure 1). The concentrations of other parameters evaluated did not differ between groups. There were no differences in the concentrations of the measured factors according to the NET grading (G1 vs. G2) (Table 6).

### 2.6. Impact of Gender on the Results of the Assays

A comparison of serum concentrations of determined factors in gender-dependent groups did not show significant differences. Values approaching significance were found for Galectin-3 concentrations, with higher concentrations observed in the female group (*p* = 0.060) (Table 7).

## 3. Discussion

The results of this study indicate that serum concentrations of selected biological factors associated with tumorigenesis may be potentially useful as biomarkers of active disease in patients with disseminated endocrine neoplasms (NET, MTC). Of all the biomarkers analyzed in our study, significant differences in TNF-α serum concentrations were observed between patients with endocrine neoplasms and healthy control groups. In the case of Fascin, there were differences between certain subtypes of endocrine tumors. These results show the potential usefulness of TNF-α and Fascin in the diagnostic and therapeutic process that facilitates the early detection of tumor recurrence after radical surgery, monitoring response to therapy, and disease progression.

### 3.1. TNF-Alpha as a Marker of Endocrine Neoplasms

Tumor necrosis factor-alpha (TNF-α) is a proinflammatory cytokine produced by various host immune cells and tumor cells [10]. Its role in carcinogenesis is complex, exhibiting both tumor promotion by stimulating cancer cell growth and proliferation, enhancing the invasive capabilities of cancer cells, facilitating metastasis, and promoting the formation of new blood vessels that supply nutrients to tumors. TNF inhibitors have the ability to induce cell death in cancer cells, demonstrating significant antitumor effects [11]. The significant increase of TNF-α serum concertation in patients with NET and MTC compared to the control group (*p* = 0.004 and *p* = 0.005, respectively) underscores its potential as a biomarker for these endocrine neoplasms. In our study among patients with NET, the concentrations of TNF-α were significantly higher in those with pancreatic NET (panNET) compared to other subtypes of NET (*p* = 0.040), suggesting an association between the location of the primary tumor and the systemic inflammatory response. In general, there are little data in the literature on the use of TNF-α in NET. The available data show that the immunohistochemical expression of TNF-α was increased in NET groups with higher proliferation rates, as well as in those with higher tumor grades. Furthermore, the immunohistochemical expression of TNF-α positively correlated with poor treatment outcomes [12]. In another study, as in ours, the serum TNF-alpha concentration was significantly higher in the NET patient group than in the healthy group [13], confirming the potential usefulness of TNF-α as a biomarker of disseminated NET. It should be noted that in our study, no significant differences in TNF-alpha concentrations were observed according to NET classification (G1 vs. G2), sex, and type of somatostatin analog used, suggesting that TNF-α may primarily reflect the presence of disease and primary tumor site rather than specific biological subtypes or therapeutic interventions.

### 3.2. Role of Fascin in Endocrine Neoplasms

Fascin is an actin-bundling protein encoded by the *FSCN1* gene that plays a key role in the organization of actin filaments in the cytoskeleton. It influences the maintenance of the cell’s structure and its ability to move. Fascin-1 is particularly important because its overexpression has been observed in various types of cancer, which may contribute to increased migration and invasiveness of cancer cells [14]. Its overexpression has been associated with a poor prognosis in various types of cancer [15,16,17,18], but it was not previously evaluated in NET. Fascine also enhances the metabolic stress- and chemo-resistance of cancer cells [8,19,20]. Our study highlights differences in Fascin concentrations between disease subtypes. Patients with MTC demonstrated significantly lower concentrations of Fascin compared to those with NET or adrenal neoplasms (*p* = 0.007 and *p* = 0.048, respectively), while the differences between NET patients and controls approached statistical significance (*p* = 0.076), suggesting a possible association of Fascin with the pathology of NET.

### 3.3. VEGF and the Impact of Somatostatin Analogs

VEGF signaling pathways in NENs represent a highly investigated area among angiogenic factors, but studies analyzing this pathway have shown contradictory results [9]. Although some research indicates elevated concentrations of VEGF in NET patients compared to healthy individuals, no clear association has been established between VEGF concentrations, tumor grading, or aggressiveness [21]. Pavel et al. explored the relationship between tumor growth and angiogenic factor release, demonstrating a reduction in VEGF concentrations after the initiation of octreotide therapy, with concentrations rising again in progressive disease cases [6]. While in our study VEGF concentrations did not differ significantly between patients and controls in general, notable differences were observed within the NET subgroup based on the type of somatostatin analog used. Patients treated with lanreotide demonstrated significantly lower concentrations of VEGF compared to those treated with octreotide (*p* = 0.035). This finding may reflect the possibility that somatostatin analogs influence VEGF expression, potentially affecting tumor vascularization and progression. However, given the small sample size, these findings should be interpreted with caution. In a prospective one-center study of NET patients treated with SSA, monitoring angiogenic factors (VEGF-R2, VEGF-R3, and VCAM-1), their serum concentrations did not help identify good responders to somatostatin analog therapy. Furthermore, there was no association between a change in serum angiogenic factors and tumor response [22].

### 3.4. Other Biomarkers

Galectins are a family of proteins that bind to β-galactosides. They play an important role in various biological processes, including the regulation of cell-cell adhesion, immune response, cell adhesion, apoptosis, and autophagy. In the context of cancer, galectins are particularly important because of their involvement in tumor progression, metastasis, and immune evasion. Among the 15 members of the lectin family, Galactin-1 and Galectin-3 appear to be the most important factors in cancer biology [23] but were not validated in NETs. The study from 2023 revealed an important mechanism in promoting Galectin-3-mediated cancer progression and metastasis in colon cancer [24]. In our study, no significant differences were found in serum concentrations of Galectin-1 or Galectin-3, between patients and controls, or between disease subtypes, except for a trend toward higher concentrations of Galectin-3 in women (*p* = 0.060). This finding could reflect gender-specific differences in tumor biology or immune responses, although more studies with larger cohorts are needed to confirm these trends.

Another important factor is fibroblast growth factor (FGF), which primarily facilitates angiogenesis, along with promoting cell growth, differentiation, and migration. It plays a crucial role in the development and progression of various tumors [25,26]. FGF contributes to cell proliferation and stromal formation, its effects being amplified by serotonin. Furthermore, FGF can stimulate the expression of genes encoding connective tissue growth factors, which are involved in the regulation of myofibroblast differentiation, collagen production, and fibroblast proliferation [27]. FGF also did not demonstrate significant differences between patients and controls, although its concentrations trended toward significance (*p* = 0.065). This suggests that although FGF can contribute to the remodeling of the tumor microenvironment, further studies with larger sample sizes are needed to determine the role of FGF in neuroendocrine tumors.

### 3.5. Limitations and Future Directions

Although this study provides valuable preliminary information, it is limited by its relatively small sample size, particularly within the subgroups of MTC and adrenal neoplasms, and the heterogeneous nature of the patient population; however, a single-center study allowed us to maintain the homogeneity of the study group in terms of genetic and environmental characteristics. Future multicenter and multiethnic samples must be performed to verify our results and to validate these findings and determine whether TNF-α and Fascin can serve as reliable early markers of disease recurrence and progression.

## 4. Conclusions

In light of the ongoing need for non-invasive and accessible diagnostic tools in endocrine oncology, our findings suggest that TNF-α and Fascin merit further investigation as potential biomarkers of tumor activity. However, these preliminary results require validation in larger, prospective, and tumor-specific studies before definitive conclusions can be drawn regarding their utility in routine monitoring of recurrence.

## Figures and Tables

**Table 1:** Demographic and clinical characteristics of the study group.

Group		No	Female/Male	Median Age (Range) Years	Dissemniation of npl (No)
Control		25	20/5	51 (26–78)	0
NET	Total	30	17/13	66 (26–86)	30
	Pancreatic NETs (panNET)	12 (1 insulinoma)			
	Small Intestine NETs (siNET)	11 (5 carcinoid syndrome)			
	Other NETs (rectum, stomach, lung)	7			
MTC		6	1/5	56 (37–65)	6
ACC		3	2/1	63 (44–89)	3
PG		4	2/2	54.5 (40–61)	4

**Table 2:** Medians and ranges of all the factors measured for the endocrine neoplasms and the healthy control groups.

Parameter	Patients Value (Range)	Control Group Value (Range)	*p*
VEGF (ng/mL)	391.90 (9.08–2100.00)	306.50 (0.00–901.40)	0.245
TNF-α (ng/mL)	2.88 (0.00–9.89)	0.93 (0.00–5.70)	0.008
Galectin-1 (ng/mL)	1.6500 (0.85–3.28)	1.64 (0.00–5.19)	0.983
Galectin-3 (ng/mL)	2.64 (1.23–6.64)	2.69 (0.92–8.06)	0.873
Fascin (ng/mL)	4.95 (0.00–100.00)	3.67 (0.00–100.00)	0.245
FGF (ng/mL)	2.2750 (0.00–23.88)	5.8300 (0.00–32.14)	0.065

**Table 3:** Medians and ranges of Fascin and TNF-α in patients divided by primary neoplasm and healthy control group.

Factor	NET Patients Value (Range) No 30	MTC Patients Value (Range) No 6	Adrenal Neoplasm Value (Range) No 7	Control Group Value (Range) No 25	*p*
Fascin (ng/mL)	5.59 (0.00–100.00)	0.52 (0.02–4.17)	5.28 (1.13–17.95)	3.67 (0.00–100.00)	0.035
TNF-α (ng/mL)	2.88 (0.00–9.89)	2.77 (1.65–5.62)	0.65 (0.00–5.14)	0.93 (0.00–5.7)	0.007

**Table 4:** Medians and ranges of all factors measured in NET patients according to the type of somatostatin analog used.

Parameter	Lanreotide Use Value (Range) No 22	Octreotide Use Value (Range) No 7	*p*
VEGF (ng/mL)	309.1000 (9.08–860.10)	488.6000 (302.70–558.30)	0.035
TNF-α (ng/mL)	2.9200 (0.00–9.89)	1.4900 (0.00–5.26)	1.000
Galectin-1 (ng/mL)	2.0050 (0.00–3.69)	1.6200 (1.39–5.19)	1.000
Galectin-3 (ng/mL)	2.8300 (1.23–5.08)	2.4500 (1.37–5.10)	1.000
Fascin (ng/mL)	5.4900 (0.00–54.52)	4.8400 (0.27–20.03)	1.000
FGF (ng/mL)	3.8150 (0.00–23.88)	0.0000 (0.00–22.65)	1.000

**Table 5:** Medians and ranges of all factors measured in NET patients in relation to the location of the main focus of the NET.

Parameter	panNET Value (Range) No 12	siNET Value (Range) No 11	Other NET Value (Range) No 7	*p*
VEGF (ng/mL)	359.4500 (9.08–860.00)	410.9000 (96.80–558.30)	306.50 (0.00–901.40)	0.753
TNF-α (ng/mL)	4.4900 (0.00–9.89)	1.4900 (0.00–6.69)	0.93 (0.00–5.70)	0.040
Galectin-1 (ng/mL)	2.2600 (0.00–5.19)	1.5700 (1.39–3.77)	1.64 (0.00–5.19)	0.218
Galectin-3 (ng/mL)	2.64 (1.23–5.08)	3.0700 (2.33–54.52)	2.69 (0.92–8.06)	0.890
Fascin (ng/mL)	2.88 (0.85–26.78)	6.6950 (2.33–54.52)	3.67 (0.00–100.00)	0.890
FGF (ng/mL)	2.530 (0.00–14.48)	0.6000 (0.00–15.02)	5.8300 (0.00–32.14)	0.890

**Table 6:** Median values and ranges of all factors measured in NET patients in relation to WHO grading (G1 vs. G2).

Parameter	G1 Value (Range) No 10	G2 Value (Range) No 18	*p*
VEGF (ng/mL)	410.9000 (96.80–558.30)	368.1000 (114.30–860.10)	1.000
TNF-α (ng/mL)	2.4500 (0.00–5.94)	2.9600 (0.00–9.89)	0.411
Galectin-1 (ng/mL)	1.6000 (1.39–5.19)	1.9700 (1.16–3.32)	1.000
Galectin-3 (ng/mL)	2.5200 (1.37–5.10)	3.1400 (1.23–5.08)	1.000
Fascin (ng/mL)	4.8400 (2.33–54.52)	5.6900 (0.00–26.78)	1.000
FGF (ng/mL)	6.3100 (0.00–22.65)	4.5300 (0.00–23.88)	1.000

**Table 7:** A comparison of serum concentrations of the determined in relation to sex.

Parameter	Females Value (Range) No 18	Males Value (Range) No 12	*p*
VEGF (ng/mL)	456.7000 (114.30–860.10)	327.0500 (9.08–558.30)	0.264
TNF-α (ng/mL)	2.1850 (0.00–7.64)	3.1800 (0.00–9.89)	1.000
Galectin-1 (ng/mL)	1.8950 (1.16–5.19)	2.0300 (0.00–3.77)	0.710
Galectin-3 (ng/mL)	3.1550 (1.23–5.08)	2.3400 (1.26–5.10)	0.060
Fascin (ng/mL)	5.6900 (0.00–54.52)	4.7550 (0.27–11.90)	1.000
FGF (ng/mL)	3.5550 (0.00–22.65)	2.5350 (0.00–23.88)	1.000

## Data Availability

The original contributions presented in this study are included in the article. Further inquiries can be directed to the corresponding author.
